# Effects of Cognitive–Motor and Motor–Motor Dual Tasks on Gait Performance in Older Adults with Sarcopenia

**DOI:** 10.3390/healthcare12121206

**Published:** 2024-06-17

**Authors:** Cenyi Wang, Baoming Jin, Aming Lu

**Affiliations:** School of Physical Education and Sports Science, Soochow University, Suzhou 215006, China; cywang11@suda.edu.cn

**Keywords:** sarcopenia, postural balance, gait, multitask

## Abstract

Background: With the advent of global aging, the health of the older population has become a critical public health challenge. The purpose of this study was to investigate the effect of dual-tasking on gait performance in patients with sarcopenia. Methods: Thirty participants with sarcopenia (age: 70.73 ± 4.12 yr, MMSE score: 26.90 ± 3.00), including 14 males and 16 females, were selected according to the diagnostic criteria of the Asian Working Group on Sarcopenia. All participants were instructed to perform the gait test in three modes: single task (ST), cognitive–motor dual task (CMDT), and motor–motor dual task (MMDT). Statistical analyses were performed using one-way ANOVA to evaluate the effects of different task types on gait parameters of the participants. Results: (1) Compared with ST walking, gait frequency, step length, and step speed decreased, and the gait cycle and double-support phase increased in patients with sarcopenia during dual-task walking (*p* < 0.05); (2) Compared with ST walking, gait variability indices such as stride frequency, stride length, and support period significantly increased in patients with sarcopenia during dual-task walking (*p* < 0.05). Conclusions: The increased difficulty in postural control caused by dual-task interference may reduce the safety of motor strategies in patients with sarcopenia and increase the risk of falls. Future studies should focus on the effects of exercise interventions on multitasking patterns in people with sarcopenia to promote balance function in these populations.

## 1. Introduction

With the increasing aging of the global population, the health of older adults has become an important challenge in the field of global public health [1]. Sarcopenia, a chronic disease closely related to aging, has a profound impact on the quality of life of the older population. Sarcopenia is characterized by a gradual decline in skeletal muscle mass and function, which not only affects the ability of elderly people to perform daily activities but also greatly increases the risk of falls due to the resulting loss of balance [2,3]. For older adults, falls can lead to fractures, disability, and even termination of life, placing a heavy burden on society and the individual. Thus, exploring the postural balance mechanism of sarcopenia, especially the influence of muscle wasting on walking, transfer, and other postures, is of great practical significance for preventing falls and enhancing the quality of life of elderly people.

At present, researchers commonly use traditional single-movement tests, such as standing on one foot, stepping on stairs with alternating feet, walking, etc., to evaluate the postural balance function of the aging population [4,5]. These methods may reflect the balance ability of the older population to a certain extent but often cannot fully simulate the complex situations faced by elderly people in real life, and the “ceiling effect” is obvious [6]. Meanwhile, in daily life, older people are often required to perform multiple tasks simultaneously, such as talking while walking and observing the environment while turning. This parallel multitasking situation places greater demands on the postural balance ability of elderly people. As a result, traditional single-task (ST) tests may not accurately assess the postural balance ability of older adults in reality. Previous studies have shown that the slow and unstable motor performance of the older population caused by aging may be closely related to a decline in cognitive functions of the brain, such as attention, executive function, abstraction, and orientation [7,8]. As a research paradigm that has evolved from neuropsychology to sport science, the dual-task (DT) approach has been increasingly applied to the assessment of balance function in the aged population in recent years. The core goal of the DT test is to introduce one or more secondary tasks (such as counting, memory, etc.) at the same time as the primary task (such as walking) to simulate the multitasking situation of older adults in real life [9]. In the DT test, older adults are expected to maintain attention and responsiveness to secondary tasks while performing the primary task. The interference of this DT may more accurately simulate the cognitive and physical load of older adults in daily life so as to more comprehensively evaluate and identify the postural control ability of the aged population [10]. Yang et al. reported that the DT test demonstrated greater sensitivity and specificity than the ST test and could more accurately identify postural balance problems in older adults [11]. Moreover, the types of dual tasks commonly used in the field of sport science include motor–motor dual task (MMDT) and cognitive–motor dual task (CMDT). In the previous studies, it was demonstrated that both types of DT tests can be used for balance testing [12], but the comparison of the effects of both types on balance function in the older population remains less reported in the literature [13].

Previous studies have shown that people with sarcopenia perform worse than normal older people on tests of dynamic balance function, suggesting that people with sarcopenia have a significant decline in dynamic postural control during the aging process [12,14]. Since people with sarcopenia suffer from reduced muscle mass and strength, performing daily activities such as walking and crossing obstacles is challenging. In DT situations, people with sarcopenia must deal with the dual challenges of physical movement and cognitive tasks simultaneously, which certainly exacerbates postural balance problems. Moreover, an increasing number of studies in recent years have shown that there may be a strong link between sarcopenia and cognitive decline [15,16]. As people age, their cognitive function gradually declines, which not only affects their ability to perform daily activities but also may complicate the symptoms of sarcopenia [17]. However, current research on the relationship between sarcopenia and cognitive decline is limited and needs to be further explored [18]. As a method to assess both physical and cognitive functions, DT testing provides a new perspective for exploring this relationship. Through the DT test, it is possible to better understand the performance of older patients with sarcopenia when both physical movement and cognitive tasks are performed concurrently, thereby further elucidating the intrinsic relationship between sarcopenia and cognitive decline.

Therefore, this study will test and compare the postural control ability of patients with sarcopenia by two modes of DT walking movements to further explore the influence of DT walking on the gait performance of patients with sarcopenia. We hope that this study can provide a useful and optimized reference for a series of clinical evaluation and rehabilitation training for patients with sarcopenia to reduce fall injuries, improve balance function, and improve quality of life.

## 2. Materials and Methods

### 2.1. Participants

Older adults with sarcopenia were recruited according to the diagnostic criteria for sarcopenia proposed by the 2019 Asian Working Group for Sarcopenia [19]. The diagnosis includes three components including muscle mass, muscle strength, and physical performance. Muscle mass was assessed by the skeletal muscle mass index (SMI), grip strength was assessed by muscle strength, and 6-m walking speed was assessed by physical activity. Individuals with low muscle mass (males: SMI ≤ 7.0 kg/m^2^, females: SMI ≤ 5.7 kg/m^2^), low muscle strength (males: grip strength < 28 kg, females: <18 kg), or low physical performance (walking speed < 1 m/s) were diagnosed with sarcopenia. This study was approved by the Ethics Committee of Soochow University (SUDA20211227H03). All participants understood the purpose and task of the study and signed the informed consent form.

### 2.2. Inclusion and Exclusion Criteria

Considering the potential factors influencing posture control in the older population, the inclusion criteria for the participants in this study were as follows: (1) Aged 65–75 years [20]; (2) Able to sit-stand transfer independently and walk through the ground; (3) Able to understand instructions and complete the test; (4) Having nearly three months without a history of medications taking musculoskeletal or nervous system medications.

The exclusion criteria for participants were as follows: (1) Had severe disease of the heart head blood vessel or severe disease such as advanced cancer; (2) Had a definite diagnosis of mental illness or cognitive dysfunction, physical disability, injury or surgery, or limb issues for nearly three months; (3) Had a position control ability for vestibular function diseases, such as vestibular neuritis, speech disorders, or central vestibular lesions; (4) Had a position control ability for visual dysfunction diseases, such as vestibular neuritis, glaucoma, macular degeneration, etc.; (5) Had artificial joints in the body or heart pacemakers and other metal device installations; (6) Had a simple Mental State Examination Scale (Mini-Mental State Examination, MMSE) score lower than 24 [21]; (7) Had an education level below the elementary school level.

### 2.3. Movement Test Protocol

**Figure 1:**
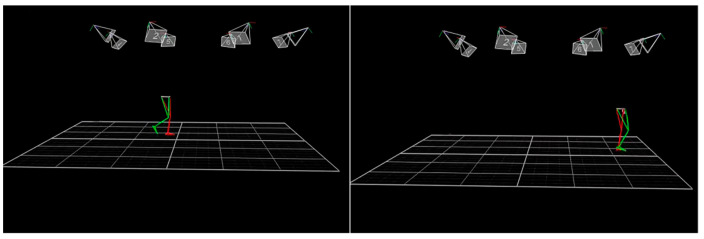
Experimental site setting.

The 3D camera for motion analysis (Vicon Motion Analysis, Yarnton, UK) consists of eight high-resolution infrared cameras (model: MX13, UK) and associated software with a resolution of 13 megapixels. In this part of the study, the sampling frequency was set to 100 Hz, and 28 infrared marker balls with a diameter of 14 mm were selected and placed on the landmark points of each limb in the lower limbs according to the human anatomical markers and the basic requirements for the 3D reconstruction of the Vicon system and in compliance with the requirements of the Plug-in-Gait model framework (see Figure 1). The specific gait test is as follows:

(1) ST walking test: Participants wore uniform black tights, with bare feet and eyes straight ahead, and walked back and forth on an 8 m long road at a daily comfortable walking speed. The duration of each test was 1 min, a total of 3 tests were performed, and the interval of each walking test was 30–60 s as a rest period [22].
(2) CMDT walking test: A counting task is performed simultaneously with a single walking task. Researchers randomly reported three-digit numbers, and participants walked while performing the continuous minus-3 task [23]. The test was performed at the same intervals as the ST walking test.
(3) MMDT walking test: A water-holding task was performed in addition to the single task walking [24]. The participants held a water cup (0.55 kg) in their right hand, and when the researcher gave the “start” command, the participants walked with the water in their hands, and the test and interval were the same as those in the ST walking test.

### 2.4. Indicator Selection

The indicators selected for analysis in this study included temporal parameters of gait, such as the gait cycle, support phase, double support phase, and swing phase percentage. The spatial parameters of gait, including step length and step width, which were standardized by using the height of the participants, were also considered. Additionally, spatiotemporal parameters, such as step speed and step frequency, were included in the analysis. In terms of gait stability, the variability index (VI) and symmetry index (SI) were identified as suitable measures.

The formula [25] for calculating the gait VI is as follows:

(1) $$VI=\frac{SD}{M}\times 100\%$$

The symbols SD and M represent the standard deviation and the average value of the relevant indicators, respectively.

The formula [26] for calculating the gait SI is as follows:

(2) $$SI=\left|\frac{2\left(L-R\right)}{L+R}\right|\times 100\%$$

The variables represented by the letters “L” and “R” are those pertinent to the left and right lower extremities, respectively. A score approaching zero on the SI indicates greater symmetry.

Behavioral parameters were used to further understand the cognitive and behavioral characteristics of the participants: correct rate (CR)—the total correct rate (%) of completion of the counting task test and reaction time (RT)—reaction to the counting task test (ms) [27].

### 2.5. Statistical Analysis

Data analysis was conducted using the IBM SPSS 22.0 statistical software program (SPSS Science, Chicago, IL, USA). One-way analysis of variance (ANOVA) was utilized to evaluate the impact of different task types (ST, CMDT, MMDT) on gait posture parameters, and *η*^2^ was used to evaluate the effect size. In the event of significant findings, post hoc multiple comparisons were employed to ascertain the distinctions between task types. All the data are presented as the mean ± standard deviation (M ± SD), and the significance level was set at α = 0.05.

## 3. Results

### 3.1. Basic Information on the Participants

A total of 54 participants with sarcopenia were recruited for this study in accordance with the diagnostic criteria. Of these, eleven were excluded due to age, five due to a history of musculoskeletal or nervous system drugs in the past three months, and eight due to MMSE scores below 24 points or education levels below the primary school level. The final 30 participants [28] with sarcopenia included 14 males and 16 females, and the sample size was calculated using G*Power software (ver. 3.1.9.2; Heinrich-Heine-Universität Düsseldorf, Düsseldorf, Germany) (N > 28). Further details can be found in Table 1.

### 3.2. Comparison of Gait Parameters in Different Tasks

The results in Table 2 show that compared with ST walking, the step frequency of sarcopenia participants in the CMDT walking was significantly decreased (*p* < 0.05), and the percentages of left and right gait cycles and the double support phase were significantly increased (*p* < 0.05). In MMDT walking, step speed was significantly slowed (*p* < 0.05), step length and step frequency were significantly decreased (*p* < 0.05), and the percentage of left/right gait cycle and double support phase were significantly increased (*p* < 0.05). No significant difference was found between CMDT and MMDT (*p* > 0.05).

### 3.3. Comparison of Gait Stability in Different Tasks

**Figure 2:**
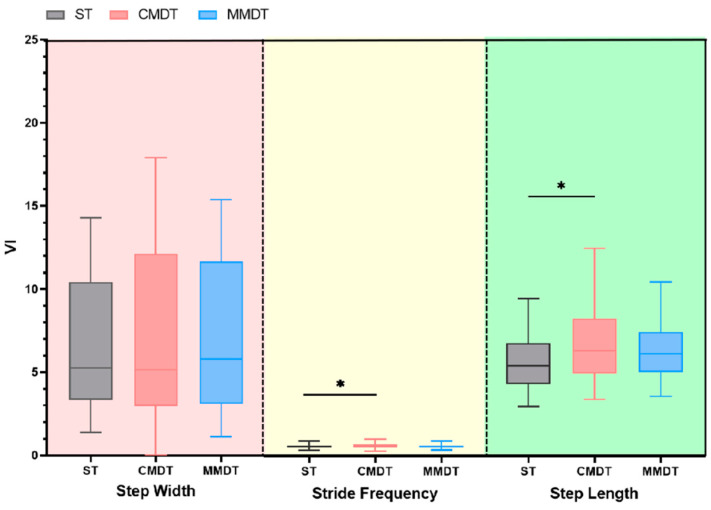
Comparison of gait variability under different tasks (*: *p* < 0.05).

**Figure 3:**
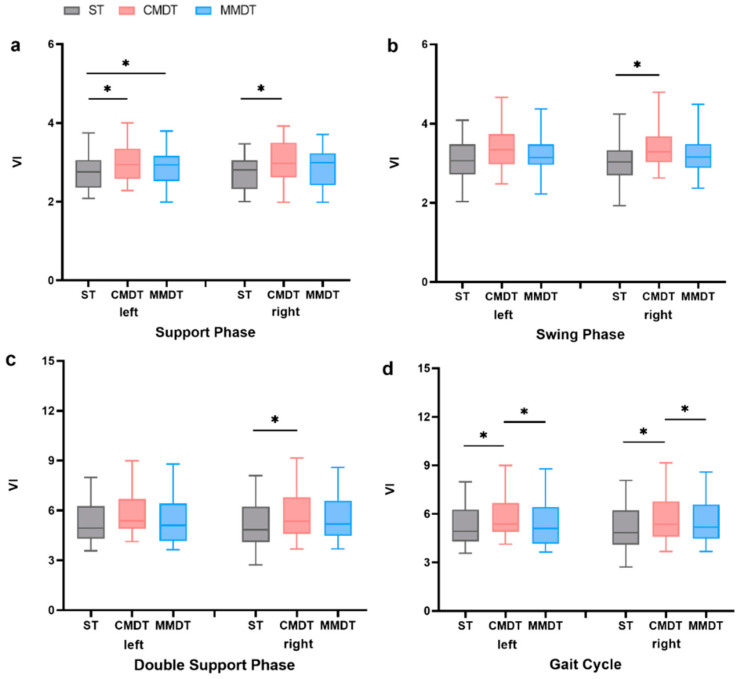
Comparison of gait cycle variability under different tasks (*: *p* < 0.05). (**a**) Comparison of support phase VI under different tasks. (**b**) Comparison of swing phase VI under different tasks. (**c**) Comparison of double support phase VI under different tasks. (**d**) Comparison of gait cycle VI under different tasks.

As shown in Figure 2, compared with ST walking, stride frequency (VI) and step length (VI) of the sarcopenia participants were significantly increased in CMDT walking (*p* < 0.05). Left and right support phase VI, right swing phase VI, right double support phase VI, and left and right gait cycle VIs were significantly increased (*p* < 0.05). In MMDT walking, the percentage of the VI in the left support phase was significantly increased (*p* < 0.05). Compared with CMDT walking, the left and right gait cycle VIs were significantly reduced in MMDT walking (*p* < 0.05); the results are described below in Figure 3.

**Figure 4:**
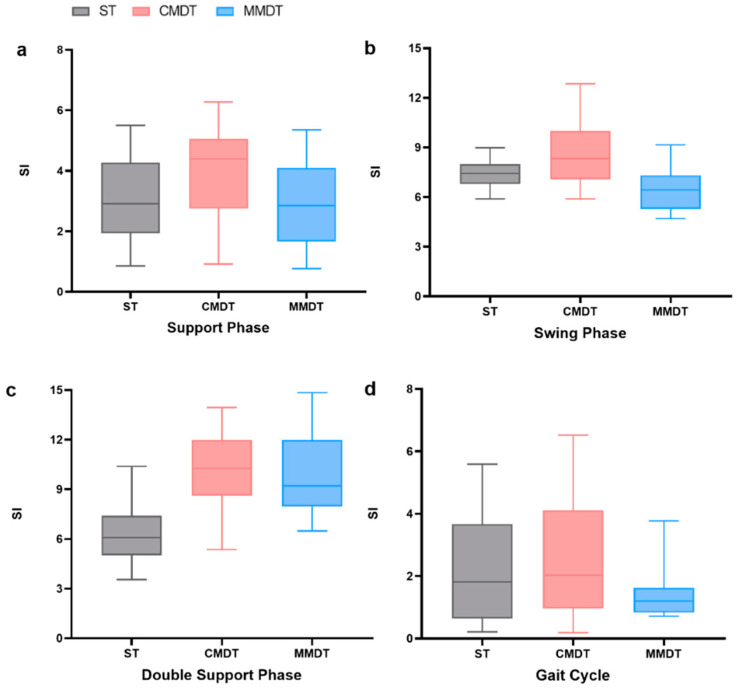
Comparison of gait symmetry under different tasks. (**a**) Comparison of support phase SI under different tasks. (**b**) Comparison of swing phase SI under different tasks. (**c**) Comparison of double support phase SI under different tasks. (**d**) Comparison of gait cycle SI under different tasks.

Moreover, in Figure 4, the participants with sarcopenia showed no significant differences in the gait SIs of ST, CMDT, and MMDT walking (*p* > 0.05).

### 3.4. Comparison of Behavioral Parameters in Different Tassks

The results of the cognitive behavioral indicators are presented in Table 3. Compared with ST walking, the CR of the participants with sarcopenia was significantly reduced under CMDT walking (*p* < 0.05). RT was significantly increased (*p* < 0.05).

## 4. Discussion

### 4.1. Effects of DTs on Gait Spatial-Temporal Performance in a Population with Sarcopenia

This study investigated the postural stability and postural control ability of people with sarcopenia during single-task walking and DT walking. The results showed that when people with sarcopenia completed the walking task, their postural stability significantly decreased, and their postural control ability significantly weakened, which verified the hypothesis of this study. As one of the key indicators used to evaluate the physical activity function and health status of the older population, walking speed is also one of the diagnostic characteristics of sarcopenia, and significant changes in the results of these studies have been reported [29]. Due to the decrease in lower limb skeletal muscle mass and strength caused by sarcopenia, the stride length and stride frequency of people with sarcopenia decrease during walking, resulting in a significant decrease in walking speed [30]. Moreover, stride length, an important parameter reflecting gait stability, is negatively correlated with gait stability; i.e., the narrower the stride length is, the worse the gait stability is [31]. As the walking speed of older adults with sarcopenia slowed significantly, the walking time increased accordingly, further compromising walking efficiency. The results of this study were also supported by the study of Mori et al. [32], who evaluated the skeletal muscle mass and gait parameters of 307 older adults and found that the duration of the support period and the double support period increased and the duration of the swing period decreased in people with sarcopenia during walking. This pattern of change is consistent with the results of this study, suggesting that people with sarcopenia tend to adopt a more stable posture to compensate for unstable gait and maintain gait stability. By increasing the duration of support, especially the double support duration (when the support surface is larger), people with sarcopenia can effectively improve postural stability during walking.

Additionally, the results of this study indicated that during dual tasking, the stride frequency of people with sarcopenia decreased, and the gait cycle and percentage of double support increased significantly. This pattern of change suggests that when elderly people perform another task at the same time while walking, their gait pattern changes significantly, and their ability to adjust gait stability is poor, which increases the risk of falling. Evidence based on EEG studies has shown that DT mode increases task-related long-range phase coherence between the parietal and frontal regions of the human brain [33,34]. Thus, when people with sarcopenia engage in another task while performing gait movement, the additional task occupies some of the attentional resources, resulting in a greater challenge for the central nervous system to rapidly integrate various sensory inputs and output the appropriate neuromuscular response. In this case, the gait strategy of reducing stride frequency and increasing gait cycle and support period time can be considered a more cautious and adaptive response [35,36].

### 4.2. Effects of DTs on Gait Stabilization in a Population with Sarcopenia

In addition to the basic temporal and spatial parameters of gait, the assessment of gait pattern variability also plays an important role. Changes in key gait parameters, such as walking speed, step length, and step frequency, are not only common predictors of fall risk but also reflect the dynamic changes in the human gait pattern mechanism [37]. The results of this study showed that the step frequency, step length, percentage of support period, percentage of swing phase, percentage of double support period, and gait cycle variation index of elderly people with sarcopenia increased significantly when they faced the cognitive DT challenge. This finding suggested that the motor–sensory control mechanism in the aged population is significantly weakened during multitasking, which may lead to a significant increase in the risk of falls. Adequate dynamic postural balance requires that the human body be free to maintain a steady or slowed gait. Such gait and postural characteristics in people with sarcopenia may be the result of a decline in the coordination of the nervous, muscular, and skeletal systems during the aging process in older people [38]. This challenge is particularly acute when dual cognitive and motor tasks need to be performed simultaneously. It is worth noting that, in contrast to the cognitive DT results, when people with sarcopenia perform motor DTs (such as water walking), they only show an increase in the variability of the left support percentage, while the variability and symmetry of other gait parameters do not change significantly. This finding suggests that gait posture control is, to some extent, an automated process. In dual motor tasks, the human body may rely predominantly on top–down neural information transmission pathways to maintain the normal operation of this automatic action, because the final action consumes relatively few attentional resources [39]. Hence, the motor DT test may not be sufficient to fully assess the variability and symmetry of gait in people with sarcopenia.

### 4.3. Effects of DTs on Cognitive–Behavioral Performance in a Population with Sarcopenia

Regarding the impact of sarcopenia on functional independence and balance function, although the decline in skeletal muscle mass and muscle strength have been widely recognized as the key factors leading to these functional declines in the elderly population, research on the impact of cognitive functions such as attention and executive function on motor function is still relatively limited, and there is insufficient evidence to support their direct relationship [40]. In this study, we compared the behavioral parameters of single and dual tasks in people with sarcopenia and found that when people with sarcopenia performed dual-counting tasks, their CR was significantly lower than that of people who performed single-counting tasks, while their RT was significantly longer. This finding demonstrated a decline in cognitive task performance in the elderly population when performing multiple tasks simultaneously, which is consistent with previous research [41,42]. The central nervous system plays a central role in the cognitive and muscular functional integrity of older adults. With aging, the central nervous system exhibits a number of age-related changes, such as the downregulation of dopaminergic neurotransmission and decreased secretion of brain-derived neurotrophic factors. These changes are often associated with declines in cognitive function, muscle strength, and athletic performance [43]. Therefore, the results of this study further support that people with sarcopenia may have impairments in cognitive and behavioral performance compared to healthy older people.

However, other studies have suggested that there is no direct association between sarcopenia and cognitive function or that this association may be influenced by other factors, such as environmental, psychological, and social factors [44]. This controversy suggests that more in-depth and comprehensive research is required on the behavior of older people with sarcopenia under cognitive load. Future studies can further explore the interaction mechanism between cognitive function and muscle function, as well as the influence of various factors on the functional activity of older people with sarcopenia, to provide a more comprehensive and scientific basis for the health management and treatment of older people with sarcopenia [45].

### 4.4. Limitation

There are several limitations in this study. First, the participants with sarcopenia included in this study were both male and female. Although some gait indicators were standardized, gender differences may increase the heterogeneity of this study. Second, although this study adopted gait parameters such as the support phase and swing phase to analyze gait performance, further analysis of lower limb interarticular kinematics may better explore the neuromuscular control characteristics of the older population. In addition, despite the age restriction of the participants included in this study, future work will further classify the age of the participants to improve the accuracy and reliability of the research results, considering the potential impact of age on postural balance in the older population.

## 5. Conclusions

In comparison to ST walking, participants with sarcopenia demonstrated shorter stride length, lower stride frequency, slower stride speed, longer gait period, and longer double-support percentage during dual-task walking. Additionally, gait variability increased, indicating a decrease in postural control and an increase in fall risk. The augmented difficulty of postural control resulting from DT interference may diminish the safety of motor strategies in participants with sarcopenia. Future research should focus on the efficacy of exercise interventions in the multitask mode in sarcopenia populations, with the goal of improving balance function in these populations.

## Figures and Tables

**Table 1:** Basic information of the participants.

N	Age (yr)	Weight (kg)	Height (cm)	BMI (kg/m^2^)	MMSE Score
30	70.73 ± 4.12	61.56 ± 9.18	161.97 ± 8.37	23.75 ± 3.15	26.90 ± 3.00

**Table 2:** Comparison of gait parameters in different tasks.

Indicators		ST	CMDT	MMDT	*F* Value	*p* Value	*η* ^2^
Step speed (m/s)		0.81 ± 0.11	0.75 ± 0.11	0.72 ± 0.10	4.965	**0.009**	0.102
Step length (%)		0.34 ± 0.02	0.33 ± 0.02	0.32 ± 0.02	3.12	**0.049**	0.067
Step width (cm)		15.17 ± 1.84	15.81 ± 2.47	15.01 ± 2.29	1.071	0.347	0.024
Stride frequency (step/min)		106.66 ± 7.00	97.86 ± 4.76	97.77 ± 4.94	23.601	**<0.001**	0.352
Gait cycle (s)	L	1.15 ± 0.09	1.25 ± 0.12	1.26 ± 0.09	10.502	**<0.001**	0.196
	R	1.16 ± 0.08	1.25 ± 0.11	1.26 ± 0.09	9.647	**<0.001**	0.182
Support phase (%)	L	62.05 ± 3.28	62.22 ± 4.01	62.44 ± 3.15	2.015	0.139	0.044
	R	62.98 ± 2.82	62.73 ± 3.58	63.09 ± 3.56	1.410	0.25	0.031
Swing phase (%)	L	37.91 ± 3.27	37.78 ± 4.05	37.23 ± 3.17	1.851	0.163	0.041
	R	37.32 ± 3.45	37.20 ± 3.60	36.91 ± 3.56	1.410	0.25	0.031
Double support phase (%)	L	23.47 ± 3.03	25.15 ± 3.94	24.87 ± 3.71	3.26	**0.043**	0.070
	R	23.85 ± 2.68	25.21 ± 3.60	26.28 ± 3.08	3.435	**0.037**	0.073

Note: *p* values in bold indicate significant differences.

**Table 3:** Comparison of behavioral parameters in different tasks.

Indicator	ST	CMDT	*F* Value	*p* Value	*η* ^2^
CR (%)	96.28 ± 3.78	89.16 ± 6.31	27.185	**<** **0.001**	0.319
RT (ms)	981.50 ± 320.06	1415.50 ± 422.97	19.415	**<** **0.001**	0.251

Note: *p* values in bold indicate significant differences.

## Data Availability

The data that support the findings of this study are available from the corresponding author upon reasonable request.
